# Cardiovascular safety of osteoanabolic agents

**DOI:** 10.1007/s00774-025-01580-4

**Published:** 2025-01-17

**Authors:** Yasuhiro Takeuchi

**Affiliations:** 1 Toranomon Hospital Endocrine Center, 2-2-2 Toranomon, Minato-ku, Tokyo, 105-8470 Japan; 2 Okinaka Memorial Institute for Medical Research, Tokyo, Japan

**Keywords:** Romosozumab, Sclerostin, Parathyroid hormone, MACE (major adverse cardiovascular events)

## Abstract

**Purpose:**

Several osteoanabolic agents have been developed to build new bone more efficiently than anti-resorptive drugs. Among them, romosozumab, an anti-sclerostin antibody, is a potent pharmacological tool to prevent fractures in osteoporosis patients. The efficacy of romosozumab in preventing osteoporotic fractures is robust. However, there remains a concern about increased cardiovascular (CV) adverse events related to romosozumab. Available data have been reviewed to address this concern.

**Methods:**

Published articles on romosozumab of which pivotal randomized controlled trials (RCTs), meta-analyses of RCTs, pharmacovigilance investigations, and retrospective observational clinical studies using real-world data were collected through PubMed and other available tools.

**Results:**

Meta-analyses of RCTs of romosozumab compared to placebo and other anti-osteoporosis drugs have left room for controversy in the CV safety of romosozumab. Investigations of the real-world data also provide no conclusive evidence in this issue.

**Conclusion:**

We need more robust evidence to establish an appropriate and reasonable guide to prescribe romosozumab in our clinical practice.

## Introduction

Osteoporosis is a metabolic disorder that affects the dynamics of bone formation and resorption to increase fracture risk. It features low bone mass and impaired bone quality, such as microarchitectural deterioration of the trabecular and cortical bone [1]. Once the integrity of bone is highly disrupted, therapeutic tools that stimulate new bone formation are necessary to prevent fragile fractures. However, standard treatments for osteoporosis are the prescription of anti-resorptive drugs that decrease bone resorption and bone formation so that their ability to restore skeletal architecture is limited [2].

Several osteoanabolic agents have been developed to overcome this limitation of anti-resorptive drugs. Intermittent subcutaneous injections of parathyroid hormone (PTH) analogs, teriparatide, an active fragment of PTH, and abaloparatide, a PTH-related protein analog, increase bone formation to demonstrate bone anabolic actions [3]. They also activate bone resorption [3], although abaloparatide is less potent in stimulating bone resorption than teriparatide [4]. Thus, their anabolic actions on bone to improve skeletal architecture are mainly derived from their activation of bone remodeling that results in a positive balance of bone formation over resorption [2, 3]. Following PTH analogs, another option of bone anabolic agent has become available to improve bone architecture by not only stimulating bone formation but inhibiting bone resorption [5]. That is a humanized antibody against sclerostin, romosozumab since sclerostin is a local factor that inhibits bone formation and stimulates bone resorption [5]. This dual-action anabolic agent has been clinically available since early 2019 in Japan and sometime later in several countries.

Cardiovascular (CV) safety concerns of romosozumab suggested in the report of the ARCH study, one of the pivotal phase 3 studies of romosozumab [6] were clinically emerging before its launch. This issue remains to be addressed during daily clinical use of romosozumab, and several observational studies have been reported [7–10]. It is difficult to uncover whether romosozumab is actually involved in the development of CV events during its use in daily clinical practice because their incidence is low. A feasible approach to answer this clinical question is to compare the incidence of CV events in osteoporotic patients at high risk of fracture treated with a bone anabolic agent, romosozumab, to that with the others, PTH analogs because they may be prescribed to patients with similar clinical backgrounds. PTH analogs are teriparatide and abaloparatide. Abaloparatide activates the same receptor for PTH, PTH1R, as teriparatide does. Thus, abaloparatide is thought to have a similar effect on the CV system to teriparatide. Teriparatide and abaloparatide both have an acute effect on circulation, which can decrease blood pressure [11] to cause dizziness although their long-term adverse effects on the CV system are not evident [11].

This article will briefly review concerns about CV safety in romosozumab based on data extracted either from prospective randomized controlled trials or from real-world clinical data for its safety.

### Potential mechanisms of the elevated CV risk with romosozumab

Mechanisms of the elevated CV risk with romosozumab have not been uncovered yet [12]. It has been reported that sclerostin may have positive, negative, or no effect on arterial calcification [13]. Sclerostin as a *SOST* gene product is most highly expressed in osteocytes, while its expression is also observed in some other tissues. The binding of sclerostin to low-density lipoprotein receptor-related proteins (LRPs) 5 and 6 impairs triggering canonical Wnt signaling in bone to decrease bone formation. Interestingly, patients with loss of LRP6 functions disrupting Wnt signaling have low bone mineral density and are susceptible to ischemic heart diseases early in their lives [14]. From these observations, activating Wnt signaling through the neutralization of sclerostin may play a role against ischemic heart diseases. In contrast, Bovijn J et al. reported that the *SOST* genetic variants were associated with a lower risk of fracture and osteoporosis and with a higher risk of myocardial infarction and/or coronary revascularization and major adverse CV events [15]. The same variants were also associated with an increased risk of type 2 diabetes mellitus, higher systolic blood pressure, and central adiposity [15]. Together, they suggested that inhibition of sclerostin might elevate CV risk, warranting a rigorous evaluation of the CV safety of romosozumab. In addition, it has been reported that decreased adipogenesis from mesenchymal stem cells following Wnt-β catenin system activation could lead to a larger deposition of free fatty acids in the arterial wall [16]. However, there are several controversies in CV safety in treatment with romosozumab. For example, a study of a similar design to Ref. [15] recently reported that genetic variants associated with lifelong reduced sclerostin expression were explored for associations with phenotypes including those related to bone physiology and CV risk factors/events in a population-based phenome-wide association study [17]. The authors concluded that natural genetic modulation of sclerostin by variants with a significant positive effect on bone physiology showed no association with lifetime risk of myocardial infarction or stroke [17]. From a mechanistic perspective, there is currently no established pathophysiologic pathway to explain such a link, although there are some putative biological effects of romosozumab on the CV system [18].

### Randomized controlled trials of romosozumab and its CV safety

Romosozumab has been approved for osteoporosis primarily based on ARCH [6] and FRAME [19] trials in postmenopausal women, where romosozumab treatment demonstrated positive results with a significant reduction in fractures. On the other hand, the ARCH trial raised safety concerns according to results of a numerical unbalance in CV events in the romosozumab group vs alendronate (50/2040, 2.5% vs 38/2014, 1.9%), ischemic cardiac events (16/2040, 0.8% vs 6/2014, 0.3%), cerebrovascular events (16/2040, 0.8% vs 7/2014, 0.3%) and adjudicated CV deaths (17/2040, 0.8% vs 12/2014, 0.6%) [6, 20] (Table 1). In contrast, the FRAME study did not show any differences in adjudicated serious CV events for romosozumab vs placebo (44/3581, 1.2% vs 41/3576, 1.1%) or in adjudicated CV deaths (17/3581, 0.5% vs 15/3576, 0.4%) [19, 20] (Table 1). One of the possible explanations for the discrepancy between ARCH and FRAME is due to different baseline profiles of CV risks [21]. Patients in ARCH were older (mean age, 74 years) than those in FRAME (mean age, 70 years). Those older than 75 years were 52.4% of participants in ARCH vs 31.2% in FRAME. The proportion of patients in ARCH (61%) with hypertension was higher than FRAME (53%), as was a previous history of CV diseases (73% vs 65%) and a history of cerebrovascular diseases (7% vs 5%). In addition, those results have raised the possibility that the discrepancy was due to a cardioprotective effect of alendronate [22]. Some observational studies suggest that bisphosphonates such as alendronate have protective effects on the development of CV events [23, 24]. However, the CV benefits of bisphosphonate therapy are not robust or consistent. For example, it has been reported that an analysis of two large, long-term, prospective databases in the US demonstrates no statistically significant differences in the long-term rates of myocardial infarction or death between bisphosphonate users and non-users [25]. The possible cardioprotective effects of bisphosphonates remain inconclusive. In addition, meta-analyses have not indicated that alendronate is protective against CV diseases [26] or overall mortality is not decreased by bisphosphonate treatment [27]. Rather, the elevated CV risk found in the ARCH study might be solely due to chance [28].

**Table 1:** Incidence of adjudicated cardiovascular serious adverse events in the first year of romosozumab trials (Ref. [20])

Category	FRAME		ARCH	
	Placebo	Romo	Alendronate	Romo
	*n* = 3576	*n* = 3581	*n* = 2014	*n* = 2040
	*n* (%)	*n* (%)	*n* (%)	*n* (%)
Adjudicated CV SAE	46 (1.3)	46 (1.3)	38 (1.9)	50 (2.5)
MACE	29 (0.8)	30 (0.8)	22 (1.1)	41 (2.0)
Cardiac ischemic event	16 (0.4)	16 (0.4)	6 (0.3)	16 (0.8)
Myocardial infarction	8 (0.2	9 (0.3)	5 (0.2)	16 (0.8)
Cerebrovascular event	11 (0.3)	10 (0.3)	7 (0.3)	16 (0.8)
Stroke	10 (0.3)	8 (0.2)	7 (0.3)	13 (0.6)
All-cause death	24 (0.7)	29 (0.8)	22 (1.1)	30 (1.5)
Cardiovascular death	15 (0.4)	17 (0.5)	12 (0.6)	17 (0.8)
Heart failure	5 (0.1)	7 (0.2)	8 (0.4)	4 (0.2)

Data are the number of women with positively adjudicated events. Data from presentations to FDA Advisory Committee (https://www.fda.gov/advisory-committees/advisory-committee-calendar/january-16-2019-meeting-bone-reproductive-and-urologic-drugsadvisory-committee-meeting-announcement)

Other randomized controlled trials also evaluated the CV safety of romosozumab. BRIDGE, including only male patients, found an increase in CV events with romosozumab compared to placebo (8/163, 4.9% vs 2/81, 2.5%) [29]. In the BRIDGE study, the participants treated with romosozumab had a higher prevalence of CV risk factors (77.3% vs 71.6%) but fewer prescriptions of cardioprotective drugs (57.1% vs 61.7%) than those in the placebo group [29]. STRUCTURE trial enrolled postmenopausal women treated either with romosozumab or teriparatide after oral bisphosphonates (218 patients per group) and found no difference in CV events between romosozumab and teriparatide groups [30].

As CV events are uncommon in clinical studies of osteoporosis, each randomized controlled trial might not have sufficient power to answer the clinical question of CV safety of romosozumab. Then, meta-analyses may be useful. A network meta-analysis of major adverse cardiovascular events (MACE) observed in randomized controlled trials of osteoporosis therapies included 5,953 women treated with romosozumab (five studies), 1,937 patients with teriparatide (six studies), and 822 with abaloparatide (one study). In this analysis, each anti-osteoporosis drug, including romosozumab, was not found to be associated with increased MACE compared to placebo, while the probabilistic surface under the cumulative ranking (SUCRA) score suggested that romosozumab was likely to be associated with CV events [31]. PTH analogs, teriparatide and abaloparatide, might be less likely associated with MACE compared to placebo [31]. The safety of romosozumab to those of teriparatide was compared in a meta-analysis of 4 randomized controlled trials (1,300 patients) that did not show significant differences in serious side effects; however, CV outcomes were not specifically assessed [32]. The other meta-analysis of 69 randomized controlled trials investigating the efficacy and safety of several osteoporosis drugs, including romosozumab and teriparatide, indicated no significant difference in serious CV events between romosozumab and teriparatide [33]. A meta-analysis comparing the efficacy and safety of denosumab to those of romosozumab suggested increased 4 point-MACE (CV death, acute myocardial infarction, stroke, and heart failure) in postmenopausal women and older men with romosozumab [34]. Other systematic reviews and meta-analyses that focused on romosozumab without comparison to other osteoporosis drugs did not find increased CV risk with the prescription of romosozumab [35, 36].

### CV safety derived from the real-world clinical data

A complementary source of evidence compiling the CV safety of romosozumab comes from real-world postmarketing reports. The earliest analysis of MACE related to romosozumab as registered in the FDA Adverse Events Reporting System (FAERS), the extensive pharmacovigilance database, between January 2019 and December 2020 identified 206 cases [7]. In that report, the event of interest was MACE (myocardial infarction, stroke, or cardiovascular death). Investigators conducted a disproportionality analysis by estimating the reporting odds ratios (RORs) and 95% confidence intervals. RORs were calculated by comparing the odds of reporting an adverse event with the drug of interest to the odds of reporting the same adverse event with other drugs. Most of the eligible cases with romosozumab were reported from Japan (*n* = 1188; 59.5%) and the US (*n* = 787; 39.4%). Among them, 206 reports of suspected MACE were identified, and most cases were either from Japan (*n* = 164; 13.8%) or the US (*n* = 41; 5.2%). ROR of MACE was elevated in general (4.07; 95% CI, 2.39–6.93). ROR of MACE in Japan (3.56; 95% CI, 1.98–6.38) was higher than the US (1.83; 95% CI, 0.84–4.00). The increase in ROR is primarily dependent on the significant disproportionality of observations in the Japanese reports, although there were no adjustments with any confounding factors in this study. Patients were older and more frequently male in the Japanese reports than those from the US. All cases with a MACE were older, and cardioprotective drugs were more frequently prescribed than those without CV events. Since elderly patients are more prone to CV diseases and because CV death is more common in men than in women, the disproportion in reports for MACE between Japan and the US may be dependent on more reports in older, male, or cardioprotective drugs taking patients from Japan than the US. An inherent bias in studies based on FAERS is that adverse effects are collected by spontaneous reporting, leading to heterogeneous reports of adverse events, without proper control groups and absolute numbers of all treated patients with romosozumab.

Another FAERS-based study of the same period focusing on the overall adverse reactions associated with romosozumab with a total of 1948 eligible events found that site reactions, heart failure, renal impairment, pneumonia, and elevated blood alkaline phosphatase could be related to romosozumab, but not acute cardiac or cerebrovascular events [8]. The third pharmacovigilance study using the Japanese Adverse Drug Event Report (JADER) database focused on CV safety in 859 patients treated with romosozumab [10]. A total of 102 ischemic heart diseases and 133 cerebrovascular events were identified. The RORs in cardiac and cerebrovascular events with romosozumab were higher than those in patients treated with bisphosphonates, denosumab, or teriparatide [10]. As the restriction not to use romosozumab in patients having recent active CV diseases was not warned at its launch in Japan, some of the patients who received romosozumab and developed MACE might have had a high-risk CV profile. Pharmacovigilance studies to identify a potential signal for elevated MACE are yet inconclusive.

### Cardiovascular concerns of romosozumab in Japan

Romosozumab was approved in January 2019 in Japan for patients who have osteoporosis with high fracture risk. It has been clinically available since March 2019, for the first time in the world. The osteoporotic patients with high fracture risk were defined to have one of the following four criteria in Japan: (i) BMD T-score ≤ − 2.5 and one or more fragility fractures, (ii) BMD T-score < − 3.3, (iii) two or more prevalent vertebral fractures, (iv) at least one grade 3 vertebral fracture [37]. A warning was issued in Japan a year after the launch of romosozumab to physicians who consider the prescription of romosozumab because there was an imbalance in the incidence of MACE between patients taking romosozumab and alendronate in the ARCH study [6]. The warning in Japan is as follows: when romosozumab is an option to treat patients with osteoporosis at high fracture risk, the benefit of fracture prevention versus the risk of CV events should be fully considered; romosozumab should not be used in patients who experienced ischemic heart disease or cerebrovascular accidents within a year. It is essentially the same as a boxed warning issued by the FDA in the United States (US).

One year after its launch, a safety report on romosozumab in Japan was issued on May 28, 2020 [38]. The report covers safety concerns after prescribing romosozumab from March 4, 2019, to March 7, 2020. The report showed that, among the overall exposure to romosozumab of 39,352 person-years, the incidence of cerebral strokes was 0.16/100 person-years, and that of ischemic cardiac diseases was 0.10/100 person-years (Table 2) [39]. Data have been updated to indicate that the incidence of cerebral strokes and ischemic cardiac diseases during each of the second, third, fourth, and fifth years is similar to that of the first year (in Japanese, https://www.pmda.go.jp/files/000269781.pdf). Those numbers are smaller than the reported incidences in Japan in the general population of cerebral strokes in the Shiga Cohort study (0.40/100 person-years) [40] and ischemic cardiac accidents in Takashima AMI Registry in Shiga Prefecture (0.17/100 person-years) [41] (Table 2). However, we must beware that the surveillance concerning about CV events using pharmacovigilance data of romosozumab might be less comprehensive than that in cohort studies as described above because those data of romosozumab were collected through passive (voluntary) surveillance. Nonetheless, the pharmacovigilance data in Japan suggest that we can appropriately manage the risk of CV accidents in patients treated with romosozumab under the observation of its boxed warnings.

**Table 2:** Cardiovascular safety of “Romosozumab Report” compared to Shiga Cohort Study in Japan

	Stroke	ICD
Romosozumab	0.164	0.1007
Shiga cohort	0.40	0.17

Incidence with 100 person-year

ICD, ischemic cardiac diseases

Another safety report was published based on the data of the Japanese Adverse Drug Event Report (JADER) database which is a pharmacovigilance database similar to the Food and Drug Administration Adverse Event Reporting System (FAERS). Registered data from April 2004 to May 2021 were extracted from the Pharmaceuticals and Medical Devices Agency website (http://www.pmda.go.jp/) and analyzed to assess the CV safety profile of romosozumab in the extensive pharmacovigilance database [10]. In that report, the outcomes of interest were CV events. Investigators conducted a disproportionality analysis by estimating the reporting odds ratios (RORs) and 95% confidence intervals. Among 859 patients with romosozumab, 102 cardiac, and 133 cerebrovascular events were identified. RORs of cardiac events (5.6, 95% CI 4.5–6.9) and cerebrovascular events (6.1, 95% CI 5.0–7.3) were elevated compared to bisphosphonates, denosumab, and teriparatide [10]. As safety warnings for serious cardiac and cerebrovascular events were reported soon after the launch of romosozumab in Japan, they might have been more detectable in romosozumab than in other drugs for osteoporosis. As for the risk of cardiac events in romosozumab users, multivariate logistic analysis identified a significant increase in the risk among patients with cardiac disease (odds ratio [OR]: 5.9, 95% confidence interval [CI] 3.5–9.9; *p* < 0.01) and hypertension (OR: 1.6, 95% CI 1.0–2.7; *p* = 0.047). In addition, the risk of cerebrovascular events in romosozumab users was significantly increased in the presence of cerebrovascular disease (OR: 2.7, 95% CI 1.2–6.2; *p* = 0.02) and hypertension (OR: 2.6, 95% CI 1.7–3.9; *p* < 0.01) [10].

Much more real-world evidence should be generated to account for sources of bias and confounding in reports of patients with cardiovascular events during the treatment with romosozumab. Until we can reach definite conclusions on the issue of CV safety of romosozumab, data so far available support the restricted prescribing recommendations in the boxed warnings that patients at a high risk of ischemic cardiac disease and stroke should not be considered for treatment with romosozumab.

### CV safety of romosozumab versus PTH analogs

Osteoanabolic agents other than romosozumab are PTH analogs, teriparatide and abaloparatide, both of which are approved only for patients with higher fracture risk, such as multiple vertebral fractures or extremely low BMD, as is romosozumab in Japan. Several factors, such as older age, smoking, and impaired renal function increase the risk of not only fracture but also CV events. According to data from the placebo group in the MORE study, osteoporotic patients with higher fracture risk, lower BMD, and/or prevalent vertebral fractures, were more likely to have CV events than those with less fracture risk [42]. It is, therefore, reasonable to evaluate the CV safety of romosozumab compared to that of PTH analogs as patients treated with either medication are likely to be susceptible to CV events associated with high fracture risk. A recent report by Stokar and Szalat has focused on this issue using the global health research network with access to anonymized data retrieved from more than 130 million individual patients, mostly from the United States and Western Europe [43]. They demonstrated that a lower risk of CV events was found in the romosozumab group than in the PTH analog group. The composite 3 point-MACE (comprised of cardiovascular death, nonfatal myocardial infarction, or nonfatal stroke) outcome was significantly less frequent in the romosozumab than PTH analog cohort (158 vs 211 patients with an outcome; *p* = 0.003), with reductions in its components: myocardial ischemic events (31 vs 58; *p* = 0.003); CV events (56 vs 79; *p* = 0.037), and cardiovascular deaths (83 vs 104; *p* = 0.099). Acute heart failure events were balanced between the cohorts (55 vs 46; *p* = 0.416) [43]. A similar pattern was observed when repeating the entire analysis in female-only cohorts. Those results suggested that in patients who were treated with any osteoanabolic agent, those with romosozumab were not exposed to a higher risk of CV events than PTH analogs. 3 point-MACE occurred significantly less frequently in women treated with romosozumab but also in all patients, suggesting that even in men use of romosozumab was not at a higher risk [43]. The results reported therein are especially reassuring in light of the FDA’s black-box warning against the use of romosozumab in patients with a cerebrovascular or CV event in the previous year as the same restriction in Japan [44]. In Europe, a stricter policy was imposed by the European Medicines Agency where even a remote history of heart attack or stroke is considered a contraindication for romosozumab [45]. More recently, a similar observational study has been reported using a Japanese administrative claims database (March 2019 to October 2022) [46]. This cohort study included 49,104 patients aged 50 years or older who initiated romosozumab (*n* = 16,125) or teriparatide (*n* = 32,979) for osteoporosis. To control for confounding due to baseline imbalances, inverse probability of treatment weighting, which targets an average treatment effect in the entire population was used in that study. Propensity scores for romosozumab initiation were calculated using a multivariable logistic regression model that included the covariates. The weighted incidence rate difference for MACE was comparable between the two drugs in Japan [46].

Osteoanabolic therapy is reserved for patients with the most severe osteoporosis, typically, with concomitant CV risk factors. Unlike outcomes based on spontaneous reporting, the study cited above [43, 46] used professionally documented ICD-10 codes from patient electronic medical records to define CV outcomes. In addition, electronic medical records were used to obtain extensive data regarding baseline CV risk, and cohorts were meticulously balanced accordingly using propensity score matching. Therefore, through analyzing data reflecting the real-world clinical evidence, the investigators might succeed in demonstrating the CV safety profile of romosozumab compared to PTH analogs. In addition, both reports of CV safety in romosozumab and PTH analogs consistently suggest that it is not worse in the former than in the latter. However, it is yet uncertain whether treatment with romosozumab is not associated with CV events because it is not prescribed to patients at high CV event risk in the US, Europe, and Japan.

## Conclusion

Romosozumab is a potent pharmacological tool to prevent fractures in osteoporosis patients, and its mechanism of action is distinct from any other drugs. The efficacy of romosozumab in preventing osteoporotic fractures is robust. However, there remains a concern about increased CV adverse events related to romosozumab. Further relevant investigations are essential to understand whether it is involved in the development of CV events or not. We need more robust evidence to establish an appropriate and reasonable guide to prescribe romosozumab in our clinical practice.
